# The Effects of* Chunghyul-Dan* (A Korean Medicine Herbal Complex) on Cardiovascular and Cerebrovascular Diseases: A Narrative Review

**DOI:** 10.1155/2016/2601740

**Published:** 2016-06-02

**Authors:** Woo-Sang Jung, Seungwon Kwon, Seung-Yeon Cho, Seong-Uk Park, Sang-Kwan Moon, Jung-Mi Park, Chang-Nam Ko, Ki-Ho Cho

**Affiliations:** Department of Cardiology and Neurology, College of Korean Medicine, Kyung Hee University, Seoul 02447, Republic of Korea

## Abstract

*Chunghyul-dan* (CHD) is a herbal complex containing 80% ethanol extract and is composed of* Scutellariae Radix*,* Coptidis Rhizoma*,* Phellodendri Cortex*,* Gardeniae Fructus*, and* Rhei Rhizoma*. We have published several experimental and clinical research articles on CHD. It has shown antilipidemic, antihypertensive, antiatherosclerotic, and inhibitory effects on ischemic stroke recurrence with clinical safety in the previous studies. The antilipidemic effect of CHD results from 3-hydroxy-3-methylglutaryl-coenzyme A (HMG-CoA) reductase and pancreatic lipase-inhibitory activity. The antihypertensive effect likely results from the inhibitory effect on endogenous catecholamine(s) release and harmonization of all components showing the antihypertensive effects. Furthermore, anti-inflammatory and antioxidant effects on endothelial cells are implicated to dictate the antiatherosclerotic effects of CHD. It also showed neuroprotective effects on cerebrovascular and parkinsonian models. These effects of CHD could be helpful for the prevention of the recurrence of ischemic stroke. Therefore, we suggest that CHD could be a promising medication for treating and preventing cerebrovascular and cardiovascular diseases. However, to validate and better understand these findings, well-designed clinical studies are required.

## 1. Introduction: Background, History, and Development

*Chunghyul-dan* (CHD) is a capsulated herbal complex, which contains an 80% ethanol extract (300 mg per capsule) composed of* Scutellariae Radix*,* Coptidis Rhizoma*,* Phellodendri Cortex*,* Gardeniae Fructus*, and* Rhei Rhizoma* (Table 1). It is also known as Daehwang-Hwang-Ryung-Haedok-Tang (Daio-orengedokuto in Japanese), which means Hwang-Ryung-Haedok-Tang (HRHT, Orengedokuto in Japanese) plus Daehwang (*Rhei Rhizoma*). In other words, CHD consists of HRHT and* Rhei Rhizoma*.

Various herbal medicines have been used to treat cardiovascular and cerebrovascular diseases such as atherosclerosis, angina, and stroke in Korean medicine clinics. HRHT, which consists of* Scutellariae Radix*,* Coptidis Rhizoma*,* Phellodendri Cortex*, and* Gardeniae Fructus*, is one of the most famous herbal medicines for treating cardiovascular and cerebrovascular diseases. The first record of the medicinal properties of the HRHT was documented in the Chinese medicine classic, “Oe-Dae-Bi-Yo (published in 752).” Traditionally, it has been used to treat pathological inflammation in gastrointestinal and cardiovascular and cerebrovascular diseases.

The modern clinical and experimental studies have also shown various evidences for the effect of HRHT on cardiovascular and cerebrovascular diseases. For instance, HRHT enhanced cerebral blood flow, decreased blood pressure, and exerted anti-inflammatory and vasodilatory effects [1]. Clinically, HRHT showed effects on abdominal obesity [2] and on the accessory symptoms of hypertension [3]. It could be suggested that these clinical effects are based on anti-inflammatory [4], antilipidemic [5], and antiplatelet effects [6] of HRHT. Furthermore, each herb in the HRHT is known to have neuroprotective, antioxidant, and antihypertensive effects [7–11]. Based on this, it was suggested that HRHT could have effects on cardiovascular disease and cerebrovascular disease.

To enhance the above-mentioned effects of HRHT, we combined HRHT and* Rhei Rhizoma*. The resulting combination medication was named as* Chunghyul-dan* (CHD), which means “purification of blood.” A previous meta-analysis [12] suggested that* Rhei Rhizoma*-based herbal preparations have significant effects on the improvement of the clinical efficacy rates, the Barthel Index, National Institutes of Health Stroke Scale, Glasgow Coma Scale, and neurological deficit scores when compared with controls using only western medicine.* Rhei Rhizoma* has been used to remove blood stasis (oehyul in Korean and oketsu in Japanese). Clinically,* Rhei Rhizoma* has been used to treat severe gastrointestinal disorders including constipation or ileus, severe inflammation such as appendicitis or pneumonia, hypertension, and cerebrovascular diseases. Furthermore,* Rhei Rhizoma* has shown neuroprotective effects on experimental ischemic stroke [13] and antilipidemic effects [14].

The process of CHD development is as follows. Herbs were extracted with 80% ethanol in boiling water for 2 hours. The extracts were filtered, evaporated with a rotary vacuum evaporator, and freeze-dried. To standardize the quality of CHD, berberine in* Coptidis Rhizoma* and* Phellodendri Cortex*, baicalin in* Scutellariae Radix*, geniposide in* Gardeniae Fructus*, and sennoside A in* Rhei Rhizoma* were quantitatively assayed according to the standardized methods [15]. To enhance dry weight yields (%) of extract, CHD was extracted with 80% ethanol instead of water. In previous studies, extracted yield of each herb with 80% ethanol was higher than extracted yields with water [16, 17]. Furthermore, we used capsulated form to improve drug compliance.

Since 2002, we have reported extensive experimental and clinical research articles related to CHD. It has shown antilipidemic, antihypertensive, antiatherosclerotic, and enhancing effects on vascular endothelial cells. Furthermore, CHD shows inhibitory effect on recurrence of ischemic stroke (small vessel occlusion type). From here onwards, this review introduces and provides the effects of CHD on cardiovascular and cerebrovascular diseases.

## 2. CHD in Dyslipidemia 

Dyslipidemia is defined as an abnormal amount of cholesterol and fat in the blood. It is known as a moderate risk factor for cardiovascular and cerebrovascular diseases such as hypertension [18], coronary artery disease [19], and ischemic stroke [20]. The conventional therapies for dyslipidemia are statins (3-hydroxy-3-methylglutaryl-coenzyme A [HMG-CoA] reductase inhibitors), fibric acid derivatives, and bile acid sequestrants. Among these, statins are the most preferred therapeutic option. Although statins are an effective medication for dyslipidemia, they have severe adverse effects such as myopathy, rhabdomyolysis, and hepatic dysfunction [21, 22]. Therefore, new types of effective and safer antilipidemic agents need to be explored.

Chung et al. [23] investigated the effects of CHD on serum lipids in patients with hyperlipidemia (see Table 2). CHD (1800 mg/day for 8 weeks) was administered to 34 patients (10 males and 24 females) with serum levels of total cholesterol, LDL cholesterol, and triglycerides higher than 200 mg/dL, 130 mg/dL, and 200 mg/dL, respectively. Follow-up lipid profile check was performed after 4 weeks (34 patients) and 8 weeks (15 patients). Four weeks later, total cholesterol and LDL cholesterol levels showed significant decrease (−8.3% (*p* < 0.05) and −7.4% (*p* < 0.05), resp.). Eight weeks later, total cholesterol and triglyceride levels showed significant decrease (−7.7% (*p* < 0.05) and −21.1% (*p* < 0.05), resp.). No serious adverse effect was observed during the follow-up. Kim et al. [24] investigated the antilipidemic effect of CHD and compared it with atorvastatin. Study design was a case-control, open-label study. The subjects were divided into 2 groups, CHD group (further subdivided into two groups based on dosage; i.e., CHD 1 group received 600 mg/day CHD and CHD 2 group received 1200 mg/day CHD) and atorvastatin group (receiving 10 mg/day atorvastatin), to investigate and identify the dose-dependent effect of CHD on hyperlipidemia. Although atorvastatin was more powerful than 600 mg or 1200 mg CHD in lowering lipid levels, both CHD 1 and CHD 2 groups showed a statistically significant lipid-lowering effect (total cholesterol (*p* < 0.05), from 268.1 ± 30.2 mg/dL to 248.6 ± 29.2 mg/dL). There was no adverse effect such as hepatic or renal toxicity during CHD treatment. However, there was no significant difference between CHD 1 and CHD 2 groups in lowering lipids. Cho et al. [25] conducted a case-control, open-label study for evaluating the therapeutic effects of CHD on hypercholesterolemia. The subjects of this study were hyperlipidemia patients whose total serum cholesterol was more than 240 mg/dL. Subjects were divided into two groups, namely, CHD group and atorvastatin group that were treated (8 weeks) with CHD (600 mg/day, *n* = 21) and atorvastatin (10 mg/day, *n* = 12), respectively. After 8 weeks, CHD showed significant lipid-lowering effect (total cholesterol (*p* < 0.01), from 269.5 ± 21.3 mg/dL to 246.9 ± 23.7 mg/dL; LDL cholesterol (*p* < 0.05), from 171.2 ± 29.8 mg/dL to 155.4 ± 26.5 mg/dL). Although the antilipidemic effect of CHD was less than atorvastatin, it was higher than historical controls from previous studies using diet therapy or placebo. During CHD treatment, there was no adverse effect on hepatic or renal toxicity.

CHD has also shown antilipidemic effect in previous experimental studies. It inhibits HMG-CoA reductase and pancreatic lipase. Kim et al. [1] assessed the HMG-CoA reductase and pancreatic lipase-inhibitory effects of CHD in hyperlipidemic model rats treated with Triton WR-1339. They showed that CHD decreased total serum cholesterol and LDL cholesterol levels in the hyperlipidemic model rats. It potently inhibited HMG-CoA reductase and pancreatic lipase, simultaneously. Therefore, they suggested that the antilipidemic effect of CHD could originate from the inhibition of pancreatic lipase and HMG-CoA reductase. Another experimental study [17] also showed that* Rhei Rhizoma,* which is a component of CHD, exerted inhibitory effects on pancreatic lipase. Flavonoid extracts from* Scutellariae Radix* such as wogonin showed antilipidemic and body weight reducing effect in mice [26, 27]. Furthermore, alkaloids from* Coptidis Rhizoma* [28, 29] and crocin isolated from* Gardeniae Fructus* [30] also revealed antihyperglycemia and antihyperlipidemia effect in experimental studies.

Therefore, we suggest that CHD could be a safe-effective medication for controlling dyslipidemia. Although the effect of CHD on dyslipidemia is lower than that of statins, it did not show any adverse effects such as myopathy or hepatic dysfunction. The mechanism of the effect of CHD on dyslipidemia can be implicated to results from its pancreatic lipase-inhibitory effects. CHD can be an alternative medication for controlling dyslipidemia in the patients with adverse effects of statins. Further studies, such as examining whether statin with CHD therapy is superior to statin only therapy, are needed to ascertain the effect of CHD on dyslipidemia and clinical use.

## 3. CHD in Hypertension

Hypertension is one of the risk factors for atherosclerotic diseases such as stroke and coronary artery disease. In a previous clinical study, HRHT (a component of CHD) exhibited therapeutic effects on abdominal obesity [2] and the accessory symptoms of hypertension [3].

We conducted a preliminary study to determine an optimal dose for antihypertensive effect of CHD [31]. In this study, 1200 mg/day CHD (twice a day, p.o., 600 mg/each time) showed short-term antihypertensive effect on stroke patients with stage 1 hypertension (systolic blood pressure 140–159 mmHg and diastolic blood pressure 90–99 mmHg).

Based on the preliminary study, Yun et al. [32] evaluated the antihypertensive efficacy of CHD on stroke in patients with stage 1 hypertension using 24-hour ambulatory blood pressure monitoring (24ABPM). Forty stroke patients with stage 1 hypertension were enrolled for the study. They were randomly assigned into two groups: CHD group and control group. Subjects in CHD group (*n* = 15) were treated with CHD (1200 mg/day) for 2 weeks, whereas control group (*n* = 13) did not receive CHD. Systolic blood pressure (SBP) of CHD group decreased from 141.37 ± 8.96 mmHg to 132.28 ± 9.46 mmHg after 2-week CHD administration (*p* = 0.03). However, SBP of control group did not show statistically significant decrease (from 138.71 ± 11.36 mmHg to 132.27 ± 8.93 mmHg). After 2 weeks of treatment, there was a significant difference in SBP between the CHD and the control groups (*p* = 0.036, Mann-Whitney *U* test). However, diastolic blood pressure and pulse rate in both groups had no significant change after treatment.

The antihypertensive effect of CHD can be explained by the findings of the following studies. HRHT and SamHwangSaShim-Tang (SHSST, Sanoushasin-to in Japanese) exert an inhibitory effect on releasing endogenous catecholamines in experimental studies [33, 34]. Another study suggested that SHSST attenuated the increase in systemic and pulmonary arterial blood pressure induced by U46619 in rats. It downregulated the expression of phosphodiesterase type 5 (PDE5), Rho-kinase (ROCK) II, and cyclooxygenase-2 (COX-2) and upregulated the expression of soluble guanylyl cyclase (sGC) alpha(1) and sGCbeta(1) in U46619 treated primary pulmonary smooth muscle cells [35]. Berberine, a main compound of* Coptidis Rhizoma*, has shown inhibitory effects on endoplasmic reticulum stress in the carotid arteries of spontaneously hypertensive rats [36]. Furthermore,* Scutellariae Radix* showed inhibitory effect on adenylate cyclase activity in experimental studies [33, 34]. In addition, crocetin (a carotenoid from* Gardeniae Fructus*) also showed protective effect against hypertension and cerebral thrombogenesis in stroke-prone spontaneously hypertensive rats [9]. Furthermore,* Rhei Rhizoma* has a depressive effect on noradrenergic and dopaminergic nerve activities [33, 34] that also may be a mechanism of antihypertensive effects of CHD.

Based on these findings, we suggest that CHD can be used as an antihypertensive agent for stage I hypertension. However, further evaluation with a larger sample size and long-term follow-up is warranted.

## 4. CHD in Endothelial Dysfunction and Atherosclerosis

The effect of CHD on atherosclerosis, especially in endothelial cell dysfunction, has been reported. Endothelial cell dysfunctions are responsible for cardiovascular diseases such as the focal localization of atherosclerotic plaque [37]. Deregulation of endothelial cell function is closely associated with the incidence of atherosclerosis. Therefore, we investigated the effect of CHD on vascular endothelial cell dysfunction. CHD exhibited antiapoptotic effects and acted as a cell-cycle-progression and cell-migration-promoting agent in a previous study [38]. Molecular studies showed that CHD activates nitric oxide synthase (NOS) mRNA, which plays an important role in the protection against atherosclerosis. It suppresses vascular cell adhesion molecule-1 (VCAM-1) mRNA, which is expressed in human endothelial cells on sites predisposed to atherosclerotic lesions [39]. Another study [40] implicated CHD in controlling a variety of inflammation related activities by regulating MCP-1 and VCAM-1 gene expression in endothelial cell. Based on this, we suggest that antiatherosclerotic effect of CHD stems from antiapoptotic, anti-inflammatory, and antioxidant effects in human vascular endothelial cells.

The effects of components of CHD on atherosclerosis have also been reported. Wogonin, an active component of* Scutellariae Radix*, revealed inhibitory effect on monocyte chemotactic protein-1 gene expression in human endothelial cells [41]. Berberine, a natural extract from* Coptidis Rhizoma*, also revealed antiatherosclerotic effect via suppression of adhesion molecule expression including vascular cell adhesion molecule-1 (VCAM-1) and intercellular adhesion molecule-1 (ICAM-1) [42] and activation of AMP-activated protein kinase (AMPK) [43].

To ascertain the clinical antiatherosclerotic effects of CHD, we investigated the effect of CHD on increased arterial stiffness using brachial-ankle pulse wave velocity (baPWV) [44]. Arterial stiffness is a contributor to the progression of atherosclerosis [45], as the increased cycle stress on the arterial walls can affect the progression. Pulse wave velocity (PWV) is a surrogate marker for atherosclerosis and is a valuable index of arterial stiffness [46]. Subjects (35) with increased baPWV (>1400 cm/sec) were enrolled for this study. All subjects were randomized and divided into 2 groups; the CHD group (*n* = 20) received 1800 mg CHD for 8 weeks and the control group (*n* = 15) was without CHD medication. After 8 weeks, baPWV was significantly decreased in the CHD group (from 1736.0 ± 271.1 cm/sec to 1599.0 ± 301.9 cm/sec, *p* = 0.032), while there was no significant change in the control (from 1668.3 ± 116.2 cm/sec to 1653.3 ± 184.1 cm/sec, *p* = 0.774). There was no clinical adverse effect.

Arterial stiffness is closely associated with atherosclerosis [46] and there is a correlation of PWV and intima-media thickness (IMT) [47]. Based on the above findings, it is suggested that CHD may prevent the progression of atherosclerosis. The mechanism of CHD mediated antiatherosclerotic effects could be the antiapoptotic and NOS activation effects on endothelial cells.

## 5. The Neuroprotective Effect of CHD in Brain Ischemia and Cerebrovascular and Neurodegenerative Diseases

Traditionally, HRHT and* Rhei Rhizoma* have been the most famous herbal preparations for stroke and ischemic brain pathology in East Asia. As mentioned above, HRHT enhances cerebral blood flow, decreases blood pressure, and exerts anti-inflammatory and vasodilatory effects [1]. Furthermore,* Rhei Rhizoma*-based herbal medicines have significant effects on the improvement of stroke as compared with controls using only western medicine in a meta-analysis [12]. Therefore, we hypothesized that CHD could also exert neuroprotective effect on brain ischemia and neurodegenerative disease.

Previous experimental studies suggested that components of CHD such as* Rhei Rhizoma* [48] and* Scutellariae Radix* [49] revealed neuroprotective and prophylactic effects on the brain ischemia of rats. Berberine, the major pharmacological active constituent of* Coptidis Rhizoma*, is also suggested to regulate neuronal apoptosis in cerebral ischemia [8]. Geniposide, a pharmacologically active component purified from* Gardeniae Fructus*, is also suggested as a suppressor of neuroinflammation through inhibiting receptor for advanced glycation end products- (RAGE-) dependent signaling pathway in Alzheimer model [50, 51].

In an experimental study [52], CHD decreased the lipopolysaccharide- (LPS-) induced expression of mRNAs encoding inducible NO synthase, tumor necrosis factor- (TNF-) alpha, interleukin-1beta, cyclooxygenase-2, and prostaglandin E2 in rat brain microglia. Furthermore, CHD significantly decreased LPS-induced phosphorylation of the ERK1/2 and p38 signaling proteins. These results suggest that CHD exerts neuroprotective effect by reducing the release of various proinflammatory molecules from activated microglia.

An experimental study using rat model of focal ischemia-reperfusion investigated the effect of CHD on ischemic brain damage [53]. CHD was administered just before reperfusion and then 2 hours after reperfusion to evaluate its neuroprotective effect. After CHD treatment, cerebral infarct volume indicated significant reduction (100, 200, and 400 mg/kg; *p* < 0.05). It also lowered microglial activation and neutrophil infiltration (*p* < 0.05). Brain-derived neurotrophic factor- (BDNF-) positive cells were significantly increased after CHD treatment (*p* < 0.05). It is thus likely that the neuroprotective mechanisms of CHD result from inhibition of microglial activation, reduction of neutrophil infiltration, and enhancement of BDNF expression. Subsequently, another study [54] showed that CHD treatment markedly decreased the cytotoxicity in 42-hour hypoxia condition and H/R condition (*p* < 0.01 and *p* < 0.05, resp.). It also significantly decreased Bax expression (*p* < 0.01) and slightly decreased Bcl-2 expression. Based on these findings, it is likely that CHD shows neuroprotective effect in N2a cells subjected to H/R by increasing the expression of the proapoptotic protein Bax.

As CHD inhibited microglial activation, the neuroprotective effects of CHD on Parkinson's disease (PD) models were also investigated [55]. In an* in vivo* study, CHD (50 mg/kg, 5 days) reduced dopaminergic neuronal damage in the substantia nigra pars compacta (SNpc) and striatum and ameliorated bradykinesia. In an* in vitro* study, CHD exhibited significant protective effects in PC12 cells by inhibiting intracellular reactive oxygen species (ROS) generation and by regulatory effects on the heme oxygenase-1 and gp91 phagocytic oxidase. Furthermore, CHD protected dopaminergic neurons in a primary mesencephalic culture against 1-methyl-4-phenylpyridinium (MPP+) neurotoxicity. These results indicated that CHD could protect neuronal cell death in PD model by inhibition of ROS generation and associated mitochondrial dysfunction.

Based on these findings, it is likely that the anti-inflammatory and antioxidant properties of CHD on brain provide the neuroprotective effect. Therefore, CHD may be used as an alternative agent for brain ischemia as well as neurodegenerative diseases in the near future. However, further clinical studies using perfusion CT to evaluate the effect of CHD in clinical set-up are required.

## 6. CHD in Prevention of Small Vessel Occlusion (SVO) Type Ischemic Stroke

As mentioned earlier, CHD has antilipidemic [17, 23–25], antihypertensive [31, 32], anti-inflammatory [52, 53], and antioxidative [54] effects and can improve endothelial cells [38–41]. It was predicted that CHD could improve and prevent the progress of microangiopathy, which is strongly associated with small vessel disease type ischemic stroke. Clinical studies were conducted to evaluate the effectiveness of CHD on the prevention of small vessel occlusion type ischemic stroke.

First, the inhibitory effect of CHD on stroke occurrence in subjects with asymptomatic SVO type ischemic stroke was investigated through observational study. For this study [56], patients who had spotty lesions (3 mm in diameter or larger) in area supplied by deep perforating artery, showing high intensity in T2 weighted (TR = 3000, TE = 80) and FLAIR images and low intensity in the T1 weighted (TR = 450, TE = 10) image were included. According to the inclusion criteria, 31 patients were recruited. 600 mg/day CHD was administered to all subjects for 1 year. Stroke occurrence and adverse effects were monitored for 1 year. Follow-up brain MRI was performed to detect new ischemic lesions 1 year after the treatment. Ten subjects dropped out and only 21 subjects completed the follow-up. Among the 21 subjects who completed follow-up, no subject experienced clinical symptoms characterized by typical stroke and no new lesions were detected in the follow-up MRI. However, among the 10 drop-out patients, 2 patients experienced ischemic stroke. The CHD administration period for drop-out patients was 2.3 ± 1.8 months.

To further understand these results, the inhibitory effect of CHD on stroke recurrence in subjects with SVO type ischemic stroke (including asymptomatic and symptomatic stroke) was investigated [57]. Seventy-three patients with SVO type ischemic stroke were treated with 600 mg/day CHD for 1 year. Among them, three patients (4.1%) experienced new ischemic stroke (symptomatic stroke = 2; asymptomatic stroke = 1).

An expansion study with a 2-year follow-up was conducted in multicenters [58]. There were 2 groups in this study: the CHD group (*n* = 148) with 600 mg/day CHD for 2 years and the control group (*n* = 208) with antiplatelet agents. New brain lesions occurred in only 3 subjects (2.0%) of the CHD group, whereas 17 subjects (8.2%) experienced stroke recurrence in the control. The OR of the CHD group for stroke recurrence was 0.232 times that of the control. Furthermore, the OR of the CHD group decreased to 0.208 when adjusted for other relevant risk factors (age, sex, antiplatelet gage medication, smoking, previous stroke, hypertension, diabetes mellitus, and hyperlipidemia). Although it was not a randomized controlled study, we suggest that the inhibitory rate of CHD on stroke recurrence is much higher than that of antiplatelet agents.

A conclusion about the effect of CHD on prevention of SVO type stroke recurrence cannot be reached due to lack of randomized controlled studies. However, from the limited data available, recurrence rate of SVO stroke in the CHD therapy group (2.0%) was lower than conventional antiplatelet therapy (6.1~12.8%) using aspirin, clopidogrel, cilostazol, triflusal, or dipyridamole [59–65]. The most common adverse effect of antiplatelet agent therapy is bleeding, which can be fatal. For instance, the overall incidence rate of hemorrhagic events was 5.58 per 1000 person-years for aspirin users and 3.60 per 1000 person-years for those without aspirin use, and the incidence rate ratio (IRR) was 1.55 (95% CI, 1.48–1.63) [66]. However, there were no serious adverse events such as bleeding during CHD treatment. Based on this, we suggest that CHD may be a safe and effective agent for prevention of SVO type ischemic stroke. We are now conducting long-term follow-up study for the effect of CHD on SVO type stroke patients to improve our understanding.

## 7. Safety and Adverse Effect

To examine the safety of CHD, a retrospective cohort review study was performed [67]. Among 656 subjects with CHD treatment, there were clinical adverse effects in 13 subjects (2.0%), 8 with gastrointestinal symptoms such as indigestion, headache, insomnia, chest discomforts, general fatigue, and thirst appearing in 1 subject, respectively. This apparent frequency of adverse effects was much lower than the safety of previous medications such as statins [68, 69]. There were no serious adverse effects such as hepatic or renal dysfunction. Furthermore, other studies on effects of CHD on dyslipidemia [23–25], hypertension [32], and arterial stiffness [44] did not show any other adverse effects. Therefore, it may be suggested that CHD is a safe medication.

## 8. Summary and Future Considerations

CHD has antilipidemic, antihypertensive, antiatherosclerotic, antioxidant, and neuroprotective effects, which could exert an inhibitory effect on microangiopathy resulting in prevention of ischemic stroke. Furthermore, several clinical trials have proven its status as a safe alternative medication. To confirm the effect of CHD, well-designed and large sized clinical studies are required to assess the potential of CHD and validate these findings.

## Figures and Tables

**Table 1:** Composition of *Chunghyul-dan*.

Constituent herbs	Scientific name (country of origin)	Weight (g)
*Scutellariae Radix*	*Scutellaria baicalensis* Georgi (Korea)	0.28
*Coptidis Rhizoma*	*Coptis japonica* Makino (Korea)	0.28
*Phellodendri Cortex*	*Phellodendron amurense* Ruprecht (Korea)	0.28
*Gardeniae Fructus*	*Gardenia jasminoides* Ellis (Korea)	0.28
*Rhei Rhizoma*	*Rheum palmatum* L. (China)	0.07

*Total*		1.2

**Table 2:** The efficacy of *Chunghyul-dan* in cardiovascular and cerebrovascular diseases in clinical studies [23–25, 32, 44, 56–58].

Disease	Author (year)	Subjects and design	Intervention	Results
Dyslipidemia	Chung et al. [23]	34 hyperlipidemia patients Before and after study	1800 mg/day CHD for 8 weeks	After 4 weeks, total cholesterol: −8.3% (*p* < 0.05), LDL cholesterol: −7.4% (*p* < 0.05) After 8 weeks, total cholesterol: −7.7% (*p* < 0.05), triglyceride: −21.1% (*p* < 0.05)
	Kim et al. [24]	62 hyperlipidemia patients Case-control, open-label study	CHD 1: 600 mg/day CHD 2: 1200 mg/day Atorvastatin: 10 mg/day for 8 weeks	After 8 weeks, in CHD 1 and CHD 2, total cholesterol: 268.1 ± 30.2 mg/dL → 248.6 ± 29.2 mg/dL (*p* < 0.05) There was no significant difference between CHD 1 and CHD 2 groups Atorvastatin was superior to 600 mg or 1200 mg CHD
	Cho et al. [56]	33 hyperlipidemia patients Case-control, open-label study	CHD: 600 mg/day Atorvastatin: 10 mg/day for 8 weeks	After 8 weeks, in CHD group, total cholesterol: 269.5 ± 21.3 mg/dL → 246.9 ± 23.7 mg/dL (*p* < 0.01), LDL cholesterol: 171.2 ± 29.8 mg/dL → 155.4 ± 26.5 mg/dL (*p* < 0.05) CHD was superior to historical controls using diet therapy or placebo Atorvastatin was superior to 600 mg CHD

Hypertension	Yun et al. [32]	28 stroke patients with stage 1 hypertension Randomized controlled, open-label study	CHD: 1200 mg/day Control: no treatment for 2 weeks	After 2 weeks, in CHD group, SBP: 141.37 ± 8.96 mmHg → 132.28 ± 9.46 mmHg (*p* = 0.03, versus control, *p* = 0.036) In control group, SBP: 138.71 ± 11.36 mmHg → 132.27 ± 8.93 mmHg (*p* > 0.05)

Atherosclerosis (arterial stiffness)	Park et al. [44]	35 subjects with increased baPWV (>1400 cm/sec) Randomized controlled, open-label study	CHD: 1800 mg/day Control: no treatment for 8 weeks	After 8 weeks, in CHD group, baPWV: 1736.0 ± 271.1 cm/sec → 1599.0 ± 301.9 cm/sec (*p* = 0.032) In control group, baPWV: 1668.3 ± 116.2 cm/sec → 1653.3 ± 184.1 cm/sec (*p* = 0.774)

Stroke prevention (SVO type)	Cho et al. [25]	31 asymptomatic ischemic stroke patients Observational study	600 mg/day CHD for 1 year	Complete follow-up patients (*n* = 21): no stroke recurrence Lost to follow-up/dropped-out patients (*n* = 10): 2 patients suffered recurrence
	Cho et al. [57]	158 ischemic stroke patients Observational study	600 mg/day CHD for 1 year	Complete follow-up patients (*n* = 73): 3 patients (4.1%) experienced stroke recurrence Lost to follow-up/dropped-out patients (*n* = 85): among 85, 54 patients were included in the final analysis → 8 patients (9.4%) had stroke recurrence OR of CHD for stroke recurrence (versus lost to follow-up): 0.12 times
	Cho et al. [58]	356 ischemic stroke patients Case-control, open-label study	CHD: 600 mg/day Antiplatelet: antiplatelet agent therapy for 2 years	In CHD group, recurrence occurred in 3 subjects (2.0%) In antiplatelet group, recurrence occurred in 17 subjects (8.2%) OR of CHD for stroke recurrence (versus antiplatelet group): 0.208 times

CHD: *Chunghyul-dan*; LDL: low-density lipoprotein; SBP: systolic blood pressure; baPWV: brachial-ankle pulse wave velocity; SVO: small vessel occlusion.
